# Sex differences in cognitive function among Chinese older adults using data from the Chinese longitudinal healthy longevity survey: a cross-sectional study

**DOI:** 10.3389/fpubh.2023.1182268

**Published:** 2023-06-29

**Authors:** Xiao Huang, Jiahui Deng, Wenbin Liu

**Affiliations:** ^1^School of Health Management, Fujian Medical University, Fuzhou, Fujian, China; ^2^Centre for Health Management and Policy Research, School of Public Health, Shandong University, Jinan, Shandong, China

**Keywords:** sex differences, cognitive function impairment, Chinese older adults, CLHLS, MMSE

## Abstract

**Objective:**

To compare the sex differences in cognitive function and its influencing factors among Chinese older adults.

**Method:**

We conducted a cross-sectional study by using data from the China Longitudinal Healthy Longevity Survey (CLHLS). According to the 32 provinces and 4 municipalities directly under the Central Government of China, 3–5 counties or districts were randomly selected in each province or city (except Tibet), and then 1–3 villages or streets were randomly selected in each county or district, from which the target population was sampled. Mini Mental State Examination (MMSE) was used to assess the cognitive function of 9,262 older adults aged 65 and above in China. Descriptive analysis was applied to demonstrate the participants’ demographic characteristics, health-related behaviors, social and non-social activity, disease status, mental and sleep condition. And then, univariate and multifactor analyses were performed to validate different risk factors for cognitive function, respectively in the general population, male older adults and female older adults.

**Result:**

The older adults with cognitive impairment accounted for 10.4% of the total population. There are significant differences in cognitive function between male and female older adults. The odds of cognitive impairment in older adult women was 1.291 times that of older adult men (OR = 1.291, 95%CI: 1.084–1.538). Among the male older adults, those who were older, highly educated, spouseless, had depressive symptoms, and lacked social activities were more likely to have cognitive impairment, whereas among the female older adults, those who were older, highly educated, and lacked social activities were more likely to have cognitive impairment.

**Conclusion:**

Overall, there are subtle differences in potential influencing factors for cognitive function between the male older adults and female older adults. Attention should be paid to the different cognitive protection measures for the older adults with different sexes.

## 1. Introduction

Cognitive function impairment is a prevalent geriatric syndrome affecting the older adults (1). Older adult individuals with mild cognitive function impairment may experience severe symptoms, and those with severe cognitive function impairment may progress to dementia or Alzheimer’s disease (AD), resulting in the inability to complete daily tasks and live independently (2). This situation is even more serious in China as it has become an aging society (3), and the number of Chinese individuals aged 60 or older is projected to reach 488 million by 2050, representing 35.6% of the total population (4). Since cognitive function impairment not only diminishes the quality of life of the older adults, but also exert burden of caregiving responsibilities on their family and the social care system (5, 6), understanding the shifting trends in cognitive function is essential for preserving social stability in an aging population.

Current research focuses primarily on analyzing demographic factors (7–9) and interpreting the effects of socioeconomic (10–12) and physiological factors on cognitive function in the older adults (13, 14). Some existing studies begin to measure the relationship between cognitive function and age and sex of the older adults. Many studies have confirmed that age and sex are important factors affecting cognition (15). In the study of healthy behaviors and lifestyles, some studies have found that smoking affects cognitive levels. Some components of nicotine and nicotine can temporarily improve cognitive function, but heavy smoking has been linked to cognitive function impairment and decline in middle age (16, 17). In addition, heavy drinking can lead to neurocognitive deficits, which can lead to mild anterograde amnesia and temporary cognitive deficits (18, 19). On the level of social participation, recently investigators have examined the effects of the degree of social isolation affects the cognition of the older adults (20, 21). Some studies have pointed out that a higher frequency of participation in social activities can help slow down the occurrence of cognitive dysfunction in the older adults (22, 23).

In terms of physical health, studies have found that physically active older adults have a lower risk of disability, functional limitations, and cognitive decline (24), and there is a negative correlation between the number of chronic diseases and cognitive decline (25). And when it comes to mental health, some studies have shown that symptoms of depression and anxiety can lead to cognitive decline (26, 27), and the quantity and quality of sleep conditions will change with the increase of age, and lack of sleep conditions will lead to decreased cognitive function (28, 29).

However, relatively few of these studies on the factors that influence cognitive function in the older adults consider sex differences. Although numerous studies have confirmed that age and sex are essential risk factors (15), sex differentiation analyses for specific categories of cognitive function are scarce. Thus, studying sex differences in cognitive function is conducive to understanding the cognitive frailty and health gap of all older adults, so that cognitive frailty can be accurately prevented and controlled based on the sex characteristics of older adults.

Therefore, this study utilized data from the 2017–2018 wave of Chinese Longitudinal Healthy Longevity Survey (CLHLS) in the present cross-sectional study. It included a large national representative sample of over 65-year-olds in determining the factors associated with cognitive function in the Chinese older adults and analyzed sex differences to increase our knowledge of cognitive health in the older adults.

## 2. Materials and methods

### 2.1. Data source

We conducted a cross-sectional study.In this study, the data set was retrieved from the Chinese Longitudinal Health and Longevity Survey (CLHLS), which was conducted by the Chinese Center for Disease Control and Prevention and directed by the Center for Healthy Aging and Development of Peking University and Duke University (30). It investigates several influential social, health, and longevity behaviors and biological and environmental risk factors (31). Initiated in 1998, the CLHLS conducted seven waves of surveys in 22 sample areas across 31 provincial administrative units from 2000 to 2018. The sample represents approximately 85% of the total population in China (32).

The questionnaire inquired about physical and mental health, lifestyle, family composition, and health care. The previous evaluation demonstrated that the CLHLS data are complete, authentic, and reliable (33).

### 2.2. Participants

Most of the participants in 2017–2018 wave of CLHLS were 65 years old and above. According to the design of this study, the inclusion criteria were older adults who completed the MMSE scale in its entirety, excluding those who were unable to complete the scale properly due to hearing and speech impairment, bedridden coma, mental illness, pathological brain injury, etc. In addition, samples with missing values in health-related behaviour, social engagement, systemic somatic diseases and mental and sleep condition were eliminated. Finally, 9,262 older adults aged 65 years and over were included in the study. More details about the participants in our study are shown in Supplementary Figure 1.

### 2.3. Measurement

#### 2.3.1. Cognitive function assessment

The Mini-Mental State Examination (MMSE) is one of the most widely used cognitive screening scales including orientation (time orientation, place orientation), memory (immediate memory, short-term memory), calculation, language (naming, repetition, listening comprehension, reading comprehension, writing), visual space, application and attention tests (34), which plays an important role in the diagnosis of cognitive impairment. Taking Chinese culture and socioeconomic development status into account, the CLHLS applied the Chinese version of MMSE to test the cognitive function of all respondents, which consists of 13 question items with total scores ranging from 0 to 30. Lower scores indicate poorer cognitive function. The validity and reliability of the Chinese MMSE has been verified (35–37). More details about the Chinese version of the MMSE are shown in Table 1.

Given that the MMSE score is susceptible to interference from educational attainment (38), this study adopt a more reasonable and accurate method to set the criterion of cognitive impairment according to different educational levels rather than setting a single standard for all participants (39, 40). If the respondents had no formal education, a score of 17 or less was considered cognitive impairment; if they had 1–6 years of education, a score of 20 or less was considered a cognitive impairment; If they had more than 6 years of education, a score of 24 or less was considered cognitive impairment; otherwise, it is considered normal cognitive function (41).

#### 2.3.2. Risk factors for cognitive impairment

In this study, risk factors for cognitive impairment in the older adults were categorized into five sets as follows:

1. Demographic characteristics, including sex, age, household registration, educational attainment and marital status;
2. Health-related behavior, including smoking, drinking and exercising;
3. Social engagement: ① “social activity,” such as Tai chi, square dancing, consort visit, other outdoor activities, playing cards or mahjong, and other social activities; ② “non-social activity,” such as housework, gardening and raising pets, reading books and newspapers, raising poultry and livestock, and watching television and listening to the radio;
4. Systemic somatic diseases: ① Circulatory system, including hypertension, heart disease and dyslipidemia; ② Endocrine system: diabetes mellitus; ③ Respiratory system: including bronchitis, emphysema, asthma, pneumonia and tuberculosis; ④ Nervous system: including stroke, cerebrovascular disease, Parkinson’s disease and epilepsy; ⑤ Urinary system: chronic nephritis; ⑥ Digestive system: including gastrointestinal ulcer, cholecystitis, cholelithiasis and hepatitis; ⑦ Exercise system: arthritis; ⑧ Immune system: rheumatism or rheumatoid arthritis; ⑨ Sensory organ: cataract or glaucoma; ⑩ Other types;
5. Mental and sleep condition: Depression scale (CESD-10) was utilized to measure the degree of depression among middle-aged and older adult individuals (42). The items of this scale include: “Do you get upset about petty things?” “Do you have a hard time concentrating?,” “Are you feeling sad or depressed?” Each scale item has four levels: “Never,” “Rarely,” “Sometimes,” and “Often or always” with scores of 0, 1, 2, and 3, respectively. For CESD-10, 12 points are the best cutoff point in China (scores≥12 points are identified as “With depressive symptoms”; scores<12 points denote “Without depressive symptoms”); While sleep condition was measured by the item “How is your sleep quality right now?” and divided into three status as “good,” “average” and “bad.”

### 2.4. Statistical analysis

In this study, categorical variables were described by frequency and percentage, and continuous variables were described by mean ± standard deviation. The cognitive function of the overall older adults were divided into two groups (normal cognitive function and impaired cognitive function) according to the MMSE scores. Comparisons of categorical information between the two groups were made using chi-square tests, whereas group comparisons of continuous information were made using t-tests. Factors with *p* < 0.05 in the univariate analysis were included in the multifactor logistic regression model, and all independent variables were involved in building the model, thus exploring the influential factors associated with cognitive impairment in the older adults. SPSS 23.0 was used to perform all of the statistical analyses. The level of statistical significance was set as *p* = 0.05.

## 3. Results

### 3.1. Characteristics of participants

The participants’ demographic data including household registration, age, marital status, educational attainment, sleep quality, smoking, drinking, exercise, cognitive ability, depression, participation in social activities, and chronic diseases, were demonstrated in Table 2. The total number of participants was 9,262, with 4,581 male and 4,681 female. In general, most older adults (70.6%) were from rural areas, with a generally low level of education. And they had very good sleep quality, and most people did not smoke (83%) or drink (83.1%), and without doing exercise (62.3%). Only a minority (10.4%) had cognitive impairment, and nearly half of the older adults had depression symptoms. Approximately 70% of older adults did not engage in social activities. More than 90% of the older adults suffered from various chronic diseases.

**Table 1:** The specific content and scoring rules of MMSE scale.

Classification	
General competence (12 marks)	Question 1: What time is it (correct = 1, incorrect or unable to answer = 0)
	Question 2: What year is it (correct = 1, incorrect or unable to answer = 0)
	Question 3: What time is the Mid-Autumn Festival (correct = 1, incorrect or unable to answer = 0)
	Question 4: What season is it (correct = 1, incorrect or unable to answer = 0)
	Question 5: Name of the district or commune where you live (correct = 1, incorrect or unable to answer = 0)
	Question 6: Name the things that can be eaten (1 mark for 1 correct answer, 7 marks for 7 or more answers)
Reactivity (3 marks)	Question 1: Name the “table” correctly (correct = 1, incorrect or unable to answer = 0)
	Question 2: Name the “apple” correctly (correct = 1, incorrect or unable to answer = 0)
	Question 3: Name the “clothes” correctly (correct = 1, incorrect or unable to answer = 0)
Attention and numeracy (6 marks)	Question 1: 20–3 =? (correct = 1, incorrect or unable to answer = 0)
	Question 2: 20–3-3 =? (correct = 1, incorrect or unable to answer = 0)
	Question 3: 20–3-3-3 =? (correct = 1, incorrect or unable to answer = 0)
	Question 4: 20–3–3-3-3-3 =? (correct = 1, incorrect or unable to answer = 0)
	Question 5: 20–3–3-3-3-3-3 =? (correct = 1, incorrect or unable to answer = 0)
	Question 6: Draw the figure on the card (correct = 1, incorrect or incomplete = 0)
Recollection skills (3 marks)	Question: Repeat “table, apple, clothes” as remembered in the “responsiveness section”(1 mark for 1 correct answer; do not count order of answers)
Language, comprehension and self-coordination skills (6 marks)	Question 1: Name the object the investigator is pointing to as “pen” (correct = 1, incorrect or unable to answer = 0)
	Question 2: Name the “watch” to which the investigator is referring (correct = 1, incorrect or unable to answer = 0)
	Question 3: Repeat the assigned sentence from the investigator (correct = 1, incorrect or unable to answer = 0)
	Question 4: Ask the respondent to hold the paper in their right hand (correct = 1, incorrect or unable to complete = 0)
	Question 5: Ask the respondent to fold the paper in half (correct = 1, incorrect or unable to complete = 0)
	Question 6: Ask the respondent to place the paper on the floor (correct = 1, incorrect or unable to complete = 0)

**Table 2:** Characteristics of the participants.

Variables	Total		Male		Female	
	*N*	Percent (%)	*N*	Percent (%)	*N*	Percent (%)
Household registration						
Urban	2,717	29.40	1,379	50.80	1,338	49.20
Rural	6,513	70.60	3,188	48.90	3,325	51.10
Age						
Young older adults (65–75)	3,080	33.30	1,603	52.00	1,477	48.00
Middle-aged older adults (76–85)	3,126	33.80	1,572	50.30	1,554	49.70
High-aged older adults (>85)	3,056	33.00	1,406	46.00	1,650	54.00
Marital status						
Married	4,650	50.60	2,908	62.50	1742	37.50
No spouse	4,539	49.40	1,638	36.10	2,901	63.90
Education						
Illiteracy	2,961	37.90	768	25.90	2,193	74.10
Elementary school and below	2,957	37.80	1744	59.00	1,213	41.00
Junior high school	999	12.80	656	65.70	343	34.30
High school	549	7.00	375	68.30	174	31.70
University and above	350	4.50	254	72.60	96	27.40
Smoking						
No	7,611	83.00	3,183	41.80	4,428	58.20
Yes	1,563	17.00	1,362	87.10	201	12.90
Drinking						
No	7,602	83.10	3,282	43.20	4,320	56.80
Yes	1,545	16.90	1,263	81.70	282	18.30
Exercising						
No	5,697	62.30	2,652	46.60	3,045	53.40
Yes	3,441	37.70	1873	54.40	1,568	45.60
Social activities						
No	6,401	69.10	3,113	48.60	3,288	51.40
Yes	2,861	30.90	1,468	51.30	1,393	48.70
Non-social activities						
No	3,197	34.50	1,515	47.40	1,682	52.60
Yes	6,065	65.50	3,066	50.60	2,999	49.40
Circulatory system						
No	297	3.20	138	46.50	159	53.50
Yes	8,965	96.80	4,443	49.60	4,522	50.40
Endocrine system						
No	1,096	11.80	491	44.80	605	55.20
Yes	8,166	88.20	4,090	50.10	4,076	49.90
Respiratory system						
No	249	2.70	120	48.20	129	51.80
Yes	9,013	97.30	4,461	49.50	4,552	50.50
Nervous system						
No	172	1.90	80	46.50	92	53.50
Yes	9,090	98.10	4,501	49.50	4,589	50.50
Urinary system						
No	231	2.50	113	48.90	118	51.10
Yes	9,031	97.50	4,468	49.50	4,563	50.50
Digestive system						
No	206	2.20	87	42.20	119	57.80
Yes	9,056	97.80	4,494	49.60	4,562	50.40
Motor system						
No	1,144	12.40	431	37.70	713	62.30
Yes	8,118	87.60	4,150	51.10	3,968	48.90
Immune system						
No	602	6.50	224	37.20	378	62.80
Yes	8,660	93.50	4,357	50.30	4,303	49.70
Receptors						
No	140	1.50	72	51.40	68	48.60
Yes	9,122	98.50	4,509	49.40	4,613	50.60
Others						
No	88	1.00	41	46.60	47	53.40
Yes	9,174	99.00	4,540	49.50	4,634	50.50
MMSE						
Normal	8,301	89.60	4,181	50.40	4,120	49.60
Impairment	961	10.40	400	41.60	561	58.40
Depression						
No	4,868	52.60	2,554	52.50	2,314	47.50
Yes	4,394	47.40	2027	46.10	2,367	53.90
Sleep						
Bad	1,376	14.90	537	39.00	839	61.00
Average	2,873	31.20	1,298	45.20	1,575	54.80
Good	4,974	53.90	2,729	54.90	2,245	45.10

There are missing values under a small number of variable entries. This part of data has been statistically processed in the original database and does not affect the overall result.

### 3.2. Differences between scores of cognitive function in male older adults and female older adults

The equations should be inserted in editable format from the equation editor. The cognitive scores using MMSE scale were compared between male and female. Table 3 shows that significant differences existed not only in total scores of MMSE cognitive, but also in general competence, reactivity, attention and numeracy, recollection skills, language, comprehension and self-coordination skills (*p* < 0.05).

**Table 3:** Differences between scores of cognitive function in male older adults and female older adults.

	Total	Male	Female	*t*	*p*
Part 1-General competence	10.25 ± 2.61	10.40 ± 2.56	10.10 ± 2.66	5.481	<0.001
Part 2-Reactivity	2.86 ± 0.51	2.88 ± 0.46	2.84 ± 0.54	3.664	<0.001
Part 3-Attention and numeracy	4.89 ± 1.54	5.23 ± 1.24	4.55 ± 1.72	21.425	<0.001
Part 4-Recollection skills	2.58 ± 0.85	2.61 ± 0.81	2.55 ± 0.87	3.173	0.002
Part 5-Language, comprehension and self-coordination skills	5.84 ± 0.57	5.87 ± 0.49	5.80 ± 0.64	5.471	<0.001
Total Score	26.26 ± 4.12	26.89 ± 3.70	25.65 ± 4.41	14.602	<0.001

### 3.3. Univariate analysis of factors influencing cognitive function in all older adults

In all older adults data (Table 4), there were significant differences between cognitively normal and cognitively impaired older adults in risk factors such as sex, age, marital status, educational attainment, sleep condition, smoking, drinking, exercising, depressive symptoms, social participation, chronic diseases of digestive system and other chronic diseases (*p* < 0.05); However, there were no significant differences between the two groups in household registration, the presence of chronic diseases such as the circulatory system, endocrine system, respiratory system, nervous system, urinary system, motor system, immune system, and receptor (*p* > 0.05).

**Table 4:** Univariate analysis of factors influencing cognitive function in all older adults.

Variables	Normal	Impairment	*χ* ^2^	*p*
Sex			26.345	<0.001
Male	4,181	400		
Female	4,120	561		
Household registration			1.563	0.211
Urban	2,418	299		
Rural	5,853	660		
Age			245.075	<0.001
Young older adults (65–75)	2,909	171		
Middle-aged older adults (76–85)	2,863	263		
High-aged older adults (>85)	2,529	527		
Marital status			101.733	<0.001
Married	4,316	334		
No spouse	3,922	617		
Education			170.216	<0.001
Illiteracy	2,466	495		
Elementary school and below	2,775	182		
Junior high school	852	147		
High school	462	87		
University and above	300	50		
Smoking			7.482	0.006
No	6,792	819		
Yes	1,431	132		
Drinking			16.132	<0.001
No	6,766	836		
Yes	1,428	117		
Exercising			19.069	<0.001
No	5,043	654		
Yes	3,145	296		
Social activities			43.906	<0.001
No	5,647	754		
Yes	2,654	207		
Non-social activities			214.374	<0.001
No	2,661	536		
Yes	5,640	425		
Chronic disease-circulatory system			3.155	0.076
No	257	40		
Yes	8,044	921		
Endocrine system			0.058	0.810
No	980	116		
Yes	7,321	845		
Respiratory system			0.150	0.699
No	225	24		
Yes	8,076	937		
Nervous system			0.634	0.426
No	151	21		
Yes	8,150	940		
Urinary system			2.361	0.124
No	200	31		
Yes	8,101	930		
Digestive system			4.947	0.026
No	175	31		
Yes	8,126	930		
Motor system			0.739	0.390
No	1,017	127		
Yes	7,284	834		
Immune system			0.116	0.734
No	542	60		
Yes	7,759	901		
Receptors			0.941	0.332
No	122	18		
Yes	8,179	943		
Others			5.822	0.016
No	72	16		
Yes	8,229	945		
Depression			33.714	<0.001
No	4,448	420		
Yes	3,853	541		
Sleep			10.763	0.005
Bad	1,202	174		
Average	2,568	305		
Good	4,495	479		

Compared with all older adults, the male older adults were equally affected by age, marital status, educational attainment, sleep condition, drinking, exercising, depressive symptoms, and social participation (*p* < 0.05); In contrast, there was a significant difference in the household registration of the male older adults (*p* < 0.05), but no significant difference in smoking and carrying all chronic diseases (*p* > 0.05) (Shown in column 5 of Table 5).

**Table 5:** Univariate analysis of factors influencing cognitive function in male older adults and female older adults.

Variables	Male				Female			
	Normal	Impairment	*χ* ^2^	*P*	Normal	Impairment	*χ* ^2^	*P*
Household registration			8.487	0.004			0.586	0.444
Urban	1,233	146			1,185	153		
Rural	2,935	253			2,918	407		
Age			66.845	<0.001			175.715	<0.001
Young older adults (65–75)	1,513	90			1,396	81		
Middle-aged older adults (76–85)	1,455	117			1,408	146		
High-aged older adults (>85)	1,213	193			1,316	334		
Marital status			32.846	<0.001			49.405	<0.001
Married	2,707	201			1,609	133		
No spouse	1,443	195			2,479	422		
Education			80.500	<0.001			80.524	<0.001
Illiteracy	664	104			1802	391		
Elementary school and below	1,644	100			1,131	82		
Junior high school	557	99			295	48		
High school	316	59			146	28		
University and above	216	38			84	12		
Smoking			0.424	0.515			1.282	0.258
No	2,900	283			3,892	536		
Yes	1,249	113			182	19		
Drinking			9.709	0.002			0.036	0.849
No	2,968	314			3,798	522		
Yes	1,179	84			249	33		
Exercising			3.913	0.048			12.940	<0.001
No	2,402	250			2,641	404		
Yes	1728	145			1,417	151		
Social activities			17.390	<0.001			25.147	<0.001
No	2,804	309			2,843	445		
Yes	1,377	91			1,277	116		
Non-social activities			72.830	<0.001			138.382	<0.001
No	1,306	209			1,355	327		
Yes	2,875	191			2,765	234		
Chronic disease- circulatory system			0.085	0.771			3.895	0.048
No	125	13			132	27		
Yes	4,056	387			3,988	534		
Endocrine system			1.891	0.169			1.014	0.314
No	440	51			540	65		
Yes	3,741	349			3,580	496		
Respiratory system			1.299	0.254			0.179	0.672
No	113	7			112	17		
Yes	4,068	393			4,008	544		
Nervous system			0.000	0.995			0.930	0.335
No	73	7			78	14		
Yes	4,108	393			4,042	547		
Urinary system			1.118	0.290			1.227	0.268
No	100	13			100	18		
Yes	4,081	387			4,020	543		
Digestive system			0.052	0.819			7.752	0.005
No	80	7			95	24		
Yes	4,101	393			4,025	537		
Motor system			0.060	0.807			0.102	0.749
No	392	39			625	88		
Yes	3,789	361			3,495	473		
Immune system			1.820	0.177			0.013	0.908
No	210	14			332	46		
Yes	3,971	386			3,788	515		
Receptors			0.293	0.588			3.328	0.068
No	67	5			55	13		
Yes	4,114	395			4,065	548		
Cancer			0.421	0.516			0.045	0.831
No	278	30			319	42		
Yes	3,903	370			3,801	519		
Others			0.000	1.000			8.260	0.004
No	37	4			35	12		
Yes	4,144	396			4,085	549		
Depression			31.200	<0.001			6.049	0.014
No	2,384	170			2064	250		
Yes	1797	230			2056	311		
Sleep			8.940	0.011			1.823	0.402
Bad	475	62			727	112		
Average	1,175	123			1,393	182		
Good	2,515	214			1980	265		

Among the female older adults, the common risk factors with the male older adults included age, marital status, educational attainment, exercise, depressive symptoms, social participation (*p* < 0.05), but there were significant differences in the chronic diseases of circulation and digestive system among the female older adults (*p* < 0.05); However, there were no significant differences in household registration, sleep condition, smoking and drinking (*p* > 0.05) (Shown in column 9 of Table 5).

### 3.4. Multifactorial analysis of factors influencing cognitive function in the older adults

Among all older adults data, sleep condition, smoking, drinking and exercising have no significant effect on cognitive ability (*p* > 0.05); The odds of cognitive impairment in female older adults was 1.291 times higher than that in male older adults (OR = 1.291, 95% CI: 1.084–1.538); Ageing had a significant effect on cognitive ability (*p* < 0.05); The odds of the older adults without a spouse suffering from cognitive impairment was 1.211 times higher than that of the married older adults (OR = 1.211, 95%CI: 1.013–1.446); Educational attainment had impacted on cognitive ability (*p* < 0.05); The older adults with depressive symptoms were 1.348 times more likely to have cognitive impairment than those without depressive symptoms (OR = 1.348, 95%CI: 1.162–1.564); Older adults who participate in social activities (Tai chi, square dancing, consort visit, other outdoor activities, playing cards or mahjong, and other social activities) were 0.766 times less likely to suffer from cognitive impairment than those who have not participated in these activities (OR = 0.766, 95%CI: 0.64–0.917); Older adults who participate in non-social activities (housework, gardening and raising pets, reading books and newspapers, raising poultry and livestock, and watching television and listening to the radio) suffer from cognitive impairment was 0.524 times lower (OR = 0.524, 95%CI: 0.445–0.618) than that of the older adults who have not participated in these activities; Digestive system and other chronic diseases had no effect on cognitive ability (*p* > 0.05) (Shown in column 2 and 3 of Table 6).

Among male older adults, household registration, sleep condition, drinking, exercising and participating in social activities had no significant effect on cognitive ability (*p* > 0.05); Ageing had a significant influence on cognitive ability in male older adults (*p* < 0.05); The unmarried male older adults were 1.351 times more likely to have cognitive impairment than married male older adults (OR = 1.351, 95%CI: 1.054–1.733);Educational attainment had impacted on cognitive ability in male older adults (*p* < 0.05); The odds of suffering from cognitive impairment for those male older adults with depressive symptoms was 1.70 times higher than that of the older adults without depressive symptoms (OR = 1.7, 95%CI: 1.35–2.139); The older adults who participate in non-social activities were 0.492 times less likely to suffer from cognitive impairment than those who have not participated in these activities (OR = 0.492, 95%CI: 0.383–0.632) (Shown in column 4 and 5 of Table 6).

**Table 6:** Multifactorial analysis of factors influencing cognitive function in older adults.

Variables^†^	Total		Male		Female	
	OR (95% CI)	*P*	OR (95% CI)	*P*	OR (95% CI)	P
Sex	1.291 (1.084, 1.538)	0.004	–	–	–	–
Household registration	–	–	1.268 (0.966, 1.663)	0.087	–	–
Age	–	<0.001	–	<0.001	–	<0.001
Middle-aged older adults (76–85)	1.645 (1.323, 2.046)	<0.001	1.507 (1.107, 2.053)	0.009	1.854 (1.363, 2.523)	<0.001
High-aged older adults (>85)	2.905 (2.306, 3.660)	<0.001	2.319 (1.665, 3.229)	<0.001	3.680 (2.647, 5.117)	<0.001
Marital status	1.211 (1.013, 1.446)	0.035	1.351 (1.054, 1.733)	0.018	1.020 (0.792, 1.314)	0.880
Education	–	<0.001	–	<0.001	–	<0.001
Elementary school and below	0.529 (0.434, 0.644)	<0.001	0.570 (0.420, 0.773)	<0.001	0.533 (0.409, 0.695)	<0.001
Junior high school	2.043 (1.609, 2.595)	<0.001	2.635 (1.858, 3.736)	<0.001	1.710 (1.189, 2.460)	0.004
High school	2.364 (1.774, 3.150)	<0.001	3.070 (2.025, 4.655)	<0.001	2.102 (1.330, 3.322)	0.001
University and above	1.709 (1.202, 2.431)	0.003	2.430 (1.487, 3.970)	<0.001	1.301 (0.684, 2.474)	0.422
Smoking	1.194 (0.955, 1.493)	0.119	–	–	–	–
Drinking	0.848 (0.676, 1.064)	0.155	0.843 (0.645, 1.100)	0.208	–	–
Exercising	0.918 (0.778, 1.082)	0.308	0.980 (0.769, 1.250)	0.872	0.916 (0.733, 1.145)	0.441
Social activities	0.766 (0.640, 0.917)	0.004	0.764 (0.581, 1.005)	0.054	0.747 (0.589, 0.949)	0.017
Non-social activities	0.524 (0.445, 0.618)	<0.001	0.492 (0.383, 0.632)	<0.001	0.554 (0.447,0.687)	<0.001
Chronic disease- Circulatory system	–	–	–	–	0.792 (0.474,1.324)	0.374
Chronic disease- Digestive system	0.834 (0.526, 1.324)	0.442	–	–	0.962 (0.736, 1.256)	0.774
Chronic disease- Other	0.564 (0.296, 1.078)	0.083	–	–	0.433 (0.194, 0.968)	0.041
Depression	1.348 (1.162, 1.564)	<0.001	1.700 (1.350, 2.139)	<0.001	1.170 (0.966, 1.417)	0.108
Sleep		0.081		0.118	–	–
Average	0.832 (0.671, 1.031)	0.093	0.810 (0.571, 1.149)	0.237	–	–
Good	0.794 (0.648, 0.972)	0.025	0.713 (0.514, 0.989)	0.043	–	–

^*^In the Hosmer and Lemeshow test of these models, *p* > 0.05.

† The control group for each variable in the above table is as follows: Sex (Male), Household registration (Urban), Age (Young older adults), Material status (Married), Education (Illiteracy), Smoking (No), Drinking (No), Exercising (No), Social activities (No), Non-social activities (No), Chronic Disease-Circulatory System (No), Chronic Diseases-Digestive System (No), Chronic Disease-Other (No), Depression (No), Sleep (Bad).

In female older adults, marital status, exercising, presence of depressive symptoms, presence of the circulatory system and digestive system chronic disease had no significant influence on cognitive ability (*p* > 0.05);Ageing had a significant influence on cognitive ability in female older adults (*p* < 0.05); Educational attainment had impacted on cognitive ability in female older adults (*p* < 0.05); Female older adults who participate in social activities was 0.747 times lower than that of the female older adults who have not participated in these activities on suffering from cognitive impairment (OR = 0.747, 95%CI: 0.589–0.949); The odds of cognitive impairment in female older adults who participate in non-social activities was 0.554 times lower than that of the female older adults who have not participated in these activities (OR = 0.554, 95%CI: 0.447–0.687); Cognitive impairment in female older adults with other chronic diseases was 0.433 times lower than that in female older adults without chronic diseases (OR = 0.433, 95%CI: 0.194–0.968) (Shown in column 6 and 7 of Table 6).

## 4. Discussion

To bridge the knowledge gap of differences in cognitive function by sex, this study used a multivariate analysis to explain the factors affecting cognitive function in older Chinese adults by parsing data from the 2017–2018 China Longitudinal Healthy Living Survey (CLHLS). From the final findings, it indicated that sex, age, marital status, and educational attainment significantly affect older adults cognitive function. And it is evident that older female with higher education at an advanced age are more likely to have cognitive impairment compared to the older male. These results not only reveal sex differences in cognitive function, but also provide practical guidance in promoting the cognitive health of this older adults group.

### 4.1. The same factors affecting cognitive function among male and female older adults

From the perspective of sex differences affecting cognition, the same factors affecting the male older adults and female older adults are: age, education attainment and social activities. Our findings indicate that the odds of cognitive dysfunction increases with age. Normal human aging can lead to a decline in cognitive function. To a physiologically healthy level, gray matter atrophy in the medial prefrontal cortex, which is associated with cognitive function, increases with age (43). The decline of brain structure and function with aging is the main cause of cognitive decline in the older adults (44).

As for the relationship between educational attainment and cognitive ability, our findings indicate that older adults with a high school diploma or higher are more likely to suffer from cognitive impairment than their illiterate counterparts, which is contrary to previous research findings that individuals with a higher level of education are more resistant to the aging process, diseases associated with aging, and brain and cognitive impairment (45, 46). However, recent studies also have pointed out that educational attainment is not associated with long-term rates of change in any cognitive domain, with studies stating that education is only associated with cognitive performance and not with cognitive decline (47). And many studies have emphasized the need to also take into account the influence of genetic factors, socioeconomic background, and socio-cultural background when analyzing the association between educational attainment and cognitive function (48–50). In light of the contradictory findings, the connection between early education and cognitive impairment in the older adults requires further investigation.

There is growing evidence that a healthy lifestyle can reduce the rate of cognitive decline with age and delay the onset of cognitive symptoms associated with age-related disorders (51). Participating in social activities is associated with improved acting ability and hand, eye, and foot coordination, among other body functions. In addition, at the molecular and cellular level, physical activity directly affects the expression of neurotransmitters and neurotrophic factors, thereby influencing synaptic plasticity as well as cellular state. Moreover, it can influence cellular inflammation and oxidative stress through hormonal regulation, which in turn affects cognition and brain health (52). And playing cards and mahjong has been shown to have a protective effect on cognitive function in the older adults (53, 54). These previous researches showing that participation in social and non-social activities protects the cognition of the older adults also supports the conclusions of our study.

### 4.2. Different influences on cognitive function between male and female older adults

In terms of the different influences on cognitive function between male and female older adults, we found that female older adults were weaker than the male older adults in each of the cognitive function scores. Studies show that decreased estrogen levels in older women are associated with cognitive dysfunction (55, 56). The decrease in estrogen will decrease blood flow to the cerebral cortex and glucose metabolism and uptake by neurons in the hippocampus, thereby increasing neuronal damage and impairing cognitive function (57). For traditional Chinese cultural and historical reasons, women in this study were significantly disadvantaged compared to men in terms of educational opportunities, nutritional intake, and occupational achievement, thereby limiting women’s cognitive maintenance and further development (58).

And simultaneously, there are sex differences in marital status and depression between male and female older adults. We found that cognitive impairment is more prevalent among male older adults without spouses than among male older adults with spouses, but marital status has no significant effect on cognitive function among female older adults. Previous research has demonstrated that family relationships are one of the most influential factors in the cognitive function of the older adults (59). A stable marriage ensures older adults receive excellent daily care and social support. Spiritual and marital support tends to be lacking in older adult individuals who are divorced or living alone, which can lead to loneliness and negative outlooks on life, resulting in psychological and cognitive impairments. Our research indicates that older men may have a greater need for family support, particularly from their partners. Those who lack such support are more likely to experience cognitive impairment (60).

Male older adults with depression symptoms were more likely (1.7 times) to have cognitive impairment than the general population (1.348 times), whereas depressive status had no effect on cognitive function in the female older adults. It indicates that the effect of depressive symptoms on cognitive dysfunction is more significant in male older adults than in female older adults. Cognitive impairment is relatively prevalent among the older adults with symptoms of depression, whereas cognitive impairment is less prevalent among the older adults who engage in social or non-social activities. There are sex differences in the structure and function of specific brain regions in depressed patients, according to research. The prefrontal limbic loop is primarily aberrant in female depressed patients, whereas the prefrontal striatum loop is primarily aberrant in male depressed patients. Moreover, the cortico-limbic striatum system abnormalities differ between male and female patients with major depression (61, 62).

This research also has some limitations. Most of the influencing factors of cognitive function involved in this study are selected based on previous studies, but our study is based on the questions in the CLHLS questionnaire. For the analysis of cognitive influencing factors in the older adults population, we only selected the cross-sectional study in 2018 based on the CLHLS database, and more samples may be needed to confirm the sex difference. The size of the questionnaire is limited, and there are many influencing factors that cannot be investigated, so the influencing factors we can select are limited. The cognitive profile of older people changes dynamically over time, however, this study is a cross-sectional study and the data is still not comprehensive. And there are various risk factors that affect cognitive profiles, but variables such as individual socio-economic base, health care conditions, experience before reaching old age, personality traits and healthy diet structure are not addressed in this study. Therefore, future studies need to further integrate tracking data from different time points, conduct longitudinal analyses of the cognitive profile of Chinese older adults and attempt to include more comprehensive variables as well as explore the interaction mechanisms of the included variables.

## 5. Conclusion

This study elucidates the factors affecting cognitive function in the older adults from the perspective of sex. There are cognitive function differences between the sexes among the older adults. Physiologically and medically, the male older adults with high education, no spouse, depressive symptoms, and lack of social activities and the female older adults with high education and lack of social activities are in a vulnerable position. In order to meet the health needs of various groups, it is necessary to develop targeted medical and social health promotion activities that consider vulnerable groups and pay attention to the gap between groups.

## Data availability statement

The datasets presented in this study can be found in online repositories. The names of the repository/repositories and accession number(s) can be found in the article/Supplementary material.

## Ethics statement

The Duke University Research Ethics Committee and Peking University approved CLHLS’s Human Subject Protection Plan. Prior to the survey, all CLHLS survey participants provided written informed consent. The patients/participants provided their written informed consent to participate in this study. Written informed consent was obtained from the individual(s) for the publication of any potentially identifiable images or data included in this article.

## Author contributions

WL and XH conceived and designed the study. XH, WL, and JD done the data acquisition, analysis, and interpretation. All authors contributed to the article and approved the submitted version.

## Funding

This study is supported by the Natural Science Foundation of Fujian Province (Grant No. 2021 J01245) and the Distinguished Young Scientific Research Talents Plan in Universities of Fujian Province (Grant No. 2018B030).

## Conflict of interest

The authors declare that the research was conducted in the absence of any commercial or financial relationships that could be construed as a potential conflict of interest.

## Publisher’s note

All claims expressed in this article are solely those of the authors and do not necessarily represent those of their affiliated organizations, or those of the publisher, the editors and the reviewers. Any product that may be evaluated in this article, or claim that may be made by its manufacturer, is not guaranteed or endorsed by the publisher.
