# Supplementary material for: Sex differences in cognitive function among Chinese older adults using data from the Chinese longitudinal healthy longevity survey: a cross-sectional study
Source: Front Public Health. 2023 Jun 29;11:1182268. doi: 10.3389/fpubh.2023.1182268 (PMC10343959; doi:10.3389/fpubh.2023.1182268)
Supplement: Supplementary file 1 [file Image_1.pdf]

Supplementary Material

Sex differences in cognitive function among Chinese older adults using data from the Chinese Longitudinal Healthy Longevity Survey (CLHLS): a cross-sectional study

Xiao Huang, Jiahui Deng , Wenbin Liu*

*** Correspondence:** Wenbin Liu: wenbinliu126@126.com

1 Supplementary Figures and Tables

1.1 Supplementary Figures

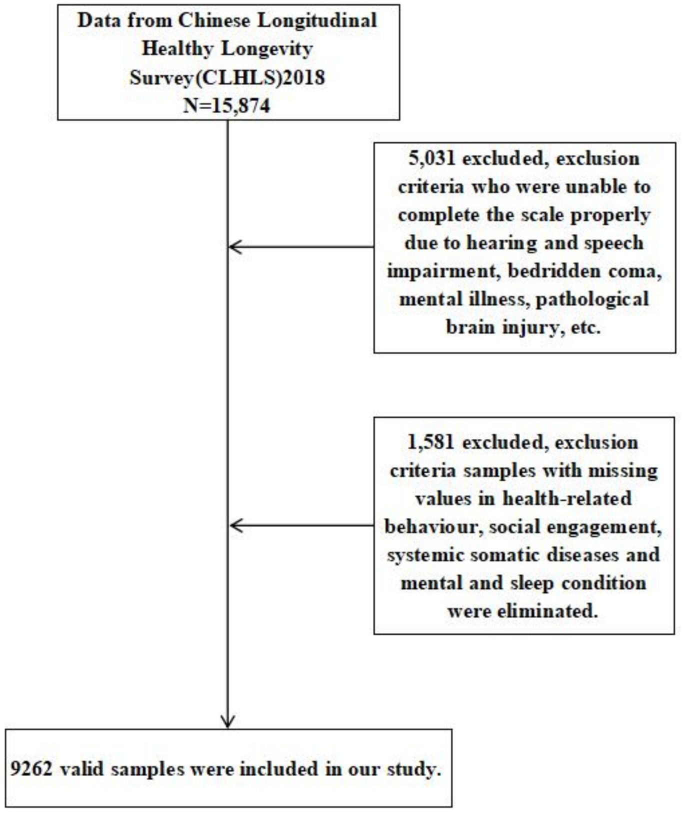

FIGURE 1
Flow diagram of participants

Supplementary Figure 1 is the flow chart of participant sample screening for this study.
